# Disentangling the Taxonomy of Rickettsiales and Description of Two Novel Symbionts (“Candidatus Bealeia paramacronuclearis” and “Candidatus Fokinia cryptica”) Sharing the Cytoplasm of the Ciliate Protist Paramecium biaurelia

**DOI:** 10.1128/AEM.02284-16

**Published:** 2016-11-21

**Authors:** Franziska Szokoli, Michele Castelli, Elena Sabaneyeva, Martina Schrallhammer, Sascha Krenek, Thomas G. Doak, Thomas U. Berendonk, Giulio Petroni

**Affiliations:** a Institut für Hydrobiologie, Technische Universität Dresden, Dresden, Germany; b Dipartimento di Biologia, Università di Pisa, Pisa, Italy; c Department of Cytology and Histology, St. Petersburg State University, St. Petersburg, Russia; d Mikrobiologie, Institut für Biologie II, Albert-Ludwigs-Universität Freiburg, Freiburg, Germany; e Indiana University, Bloomington, Indiana, USA; f National Center for Genome Analysis Support, Bloomington, Indiana, USA; University of Bayreuth

## Abstract

In the past 10 years, the number of endosymbionts described within the bacterial order Rickettsiales has constantly grown. Since 2006, 18 novel Rickettsiales genera inhabiting protists, such as ciliates and amoebae, have been described. In this work, we characterize two novel bacterial endosymbionts from Paramecium collected near Bloomington, IN. Both endosymbiotic species inhabit the cytoplasm of the same host. The Gram-negative bacterium “Candidatus Bealeia paramacronuclearis” occurs in clumps and is frequently associated with the host macronucleus. With its electron-dense cytoplasm and a distinct halo surrounding the cell, it is easily distinguishable from the second smaller symbiont, “Candidatus Fokinia cryptica,” whose cytoplasm is electron lucid, lacks a halo, and is always surrounded by a symbiontophorous vacuole. For molecular characterization, the small-subunit rRNA genes were sequenced and used for taxonomic assignment as well as the design of species-specific oligonucleotide probes. Phylogenetic analyses revealed that “Candidatus Bealeia paramacronuclearis” clusters with the so-called “basal” Rickettsiales, and “Candidatus Fokinia cryptica” belongs to “Candidatus Midichloriaceae.” We obtained tree topologies showing a separation of Rickettsiales into at least two groups: one represented by the families Rickettsiaceae, Anaplasmataceae, and “Candidatus Midichloriaceae” (RAM clade), and the other represented by “basal Rickettsiales,” including “Candidatus Bealeia paramacronuclearis.” Therefore, and in accordance with recent publications, we propose to limit the order Rickettsiales to the RAM clade and to raise “basal Rickettsiales” to an independent order, Holosporales ord. nov., inside Alphaproteobacteria, which presently includes four family-level clades. Additionally, we define the family “Candidatus Hepatincolaceae” and redefine the family Holosporaceae.

**IMPORTANCE** In this paper, we provide the characterization of two novel bacterial symbionts inhabiting the same Paramecium host (Ciliophora, Alveolata). Both symbionts belong to “traditional” Rickettsiales, one representing a new species of the genus “Candidatus Fokinia” (“Candidatus Midichloriaceae”), and the other representing a new genus of a “basal” Rickettsiales. According to newly characterized sequences and to a critical revision of recent literature, we propose a taxonomic reorganization of “traditional” Rickettsiales that we split into two orders: Rickettsiales sensu stricto and Holosporales ord. nov. This work represents a critical revision, including new records of a group of symbionts frequently occurring in protists and whose biodiversity is still largely underestimated.

## INTRODUCTION

Symbiosis is a widespread phenomenon in ciliates, and various members of both prokaryotic domains, Bacteria and Archaea, are found to populate ciliated protists (1–5).

The majority of symbiotic associations are obligate for the endosymbiont, but not for the host. More than 60 bacterial endosymbionts have been detected in various species of the ciliate genus Paramecium (6, 7). Although this number refers mainly to microscopic observations, more and more species characterizations include additional molecular descriptions as a basic feature (6, 7). The majority of these bacterial endosymbionts of ciliates belong to the order Rickettsiales, among Alphaproteobacteria (6). At present, the taxonomy of the order Rickettsiales (8, 9) includes three strongly supported monophyletic families (Rickettsiaceae, Anaplasmataceae, and “Candidatus Midichloriaceae”) and several clades formerly represented by the family Holosporaceae (“basal Rickettsiales”). In the past years, the molecular characterization of novel endosymbiotic species and the increasing genomic and metagenomic data have changed our view of this order. More complex analyses based on different genetic markers (10–12) indicate that the position of the “basal Rickettsiales” should be carefully revised. Two contrary models for the phylogeny of this group have recently arisen. One follows the historical path, in which Rickettsiales includes the families Rickettsiaceae, Anaplasmataceae, the novel described “Candidatus Midichloriaceae,” and Holosporaceae, together with related sequences (“basal Rickettsiales”). This model is usually supported by phylogenetic analyses based on prokaryotic small-subunit (SSU) rRNA gene sequences (12–19). The opposing model does not recover the monophyly of all Rickettsiales and separates “basal Rickettsiales” to form a new order. Ferla and colleagues (11) named this group Holosporales, based on analyses with prokaryotic SSU and large-subunit (LSU) rRNA gene sequences, and other studies support this notion (10, 12). We address this issue in the Discussion.

In many described unicellular symbiotic systems, a host harbors only a single endosymbiont. Nevertheless, ciliates provide several ecological niches within their unicellular body as habitats for endosymbionts. Examples are the cytoplasm, the macro- and micronuclei, the perinuclear space, and in rare cases even mitochondria (2, 7, 20), all representing closed and stable environments for colonization. Thus, multiple infections of two or more endosymbionts have been detected in different or even the same cell compartments (16, 20–24). In this work, we provide data on a so-far-stable double infection of Paramecium biaurelia collected near Bloomington, IN (USA). We characterized these two novel bacterial endosymbionts by using molecular and ultrastructural methods. In accordance with the guidelines of prokaryotic nomenclature, we propose the names “Candidatus Bealeia paramacronuclearis” and “Candidatus Fokinia cryptica.” A second Paramecium isolate harboring only “Candidatus Bealeia paramacronuclearis” was sampled nearby and was used for comparative analyses. According to our phylogenetic analyses of “basal Rickettsiales,” we propose a taxonomic reorganization of the order Rickettsiales by excluding all “basal Rickettsiales” and grouping them in the novel order Holosporales ord. nov., as suggested by Ferla and colleagues (11). We consequently propose a redefinition of the family Holosporaceae and the establishment of a new family, “Candidatus Hepatincolaceae.”

## MATERIALS AND METHODS

### Host isolation, cultivation, and identification.

The double-infected Paramecium isolate US_Bl 11III1 was derived from a freshwater sample of a small pond in the Miller Showers Park in Bloomington, IN (39°10′46.1″N, 86°32′03.6″W) in 2011. The single-infected isolate US_Bl 15I1 was sampled from approximately 17 km away, in the 0.54-km^2^ Yellowwood Lake, IN (39°11′30.2″N, 86°20′30.9″W) in 2011. Monoclonal mass cultures were established and maintained in 0.25% Cerophyl medium inoculated with Raoultella planticola (modified according to methods described by Krenek et al. [25]). Total DNA was extracted for molecular characterization of host and endosymbionts; approximately 50 Paramecium cells were washed several times in sterile spring water, incubated at room temperature overnight, and washed again to minimize bacterial contaminants. Cells were fixed in 70% ethanol, and DNA was extracted using the NucleoSpin plant DNA extraction kit (Macherey-Nagel GmbH & Co. KG, Düren, NRW, Germany), following the cetyltrimethylammonium bromide-based protocol for fungi.

Host species were identified using morphological features (26) and confirmed by sequencing the eukaryotic SSU rRNA gene (following the approach described by Petroni et al. [27]), the mitochondrial cytochrome *c* oxidase subunit 1 (COI) gene (28), and the internal transcribed spacer (ITS; modified according to methods described by Boscaro et al. [29]). PCR products were purified with the NucleoSpin gel and PCR cleanup kit (Macherey-Nagel GmbH & Co. KG, Düren, NRW, Germany) and directly sequenced at GATC Biotech AG (Constance, Germany). For sequencing, internal primers were used for the eukaryotic SSU rRNA gene (30) and ITS regions (29). The COI gene was sequenced directly in both directions, with the same primers used for amplification (28).

### Molecular characterization of the endosymbionts.

Prokaryotic SSU rRNA gene sequences of the endosymbiotic species inhabiting isolates US_Bl 11III1 and US_Bl 15I1 were obtained by direct sequencing. Touchdown PCR (31) was carried out with the Alphaproteobacteria-specific forward primer 16Sα_F19b and the Bacteria-specific reverse primer 16S_R1522a (32), as described by Szokoli and colleagues (33). PCR products were purified with the EuroGold CyclePure kit (EuroClone S.p.A. Headquarters and Marketing, Pero, Milan, Italy) and sequenced using internal primers (32).

Two different approaches, cloning and direct sequencing, were used to obtain the prokaryotic SSU rRNA gene sequence of the second endosymbiont in isolate US_Bl 11III1. For cloning, PCR amplification was carried out with Phusion high-fidelity DNA polymerase (New England BioLabs Inc., Ipswich, MA, USA). The universal primers Bac16SFor and Bac16SRev (34) were used in a touchdown PCR protocol (annealing at 58°C for 10 cycles followed by annealing at 54°C for 25 cycles). PCR products were then purified, and 3′ A overhangs were added through incubation in a reaction mix containing 1.67× Colorless GoTaq reaction buffer (Promega, Fitchberg, WI, USA) with 66.67 μM dATPs, 5 mM MgCl_2_, and 1 U GoTaq polymerase (Promega) in a total volume of 15 μl for 20 min at 72°C. This DNA was cloned into the pGEM-T vector system (Promega) and transformed into competent TOP10 Escherichia coli cells following the manufacturer's instructions. Three clones derived from DNA of US_Bl 11III1, showing identical restriction patterns after digestion with MboI (New England BioLabs Inc., Ipswich, MA, USA) but different from restriction patterns of food bacteria and the endosymbiont of the single-infected host (strain US_Bl 15I1), were sequenced from both directions using M13 primers. The resulting three sequences were used to produce a consensus sequence for the second endosymbiont isolate, US_Bl 11III1.

In order to confirm the obtained SSU rRNA gene sequence, direct sequencing was performed on the same PCR product employed for cloning, using specific internal primers Fokinia_F434 (5′-CTCTTTTGGTAGGGATGATAAT-3′), Fokinia_R434 (5′-ATTATCATCCCTACCAAAAGAG-3′), Fokinia_F1250 (5′-AGAAGGCTGCAACAGGGT-3′), and Fokinia_R1250 (5′-ACCCTGTTGCAGCCTTCT-3′).

### Fluorescence *in situ* hybridization.

In order to verify that the obtained SSU rRNA genes were derived from the endosymbionts, specific molecular probes were designed. For the endosymbiont present in both isolates, the species-specific probe Bealeia_1245 (5′-CCTATTGCTTCCTTTTGTCAC-3′; Cy3 labeled at the 5′ end; melting temperature [*T_m_*], 55.9°C) was designed. The species-specific probe FokCry_198 (5′-CTCGCAGTAACATTGCTGC-3′; Cy3 labeled at the 5′ end; *T_m_* of 56.7°C) was designed for the second endosymbiont present only in isolate US_Bl 11III1. Both probes were obtained from Eurofins Genomics GmbH (Ebersberg, Germany). Fluorescence *in situ* hybridization (FISH) was performed with each newly designed probe, in combination with EUB338 (fluorescein isothiocyanate [FITC] labeled at the 5′ end) (35), according to methods described by Szokoli and coworkers (33). Both probes were tested with formamide concentrations ranging from 0 to 50%. Their specificity was determined *in silico* by using the TestProbe Tool 3.0 (SILVA rRNA database project) (36) and the probe match tool of the Ribosomal Database Project (RDP) (37), allowing 0, 1, or 2 mismatches (Table 1).

**TABLE 1:** *In silico* matching of the species-specific probes Bealeia_1245 and FokCry_198 against bacterial SSU rRNA gene sequences^*a*^

Species-specific probe	No. of hits in database with indicated no. of mismatches					
	RDP			SILVA		
	0 mism	1 mism	2 mism	0 mism	1 mism	2 mism
Bealeia_1245	0	179	321	0	38	62
FokCry_198	0	0	16	0	0	2

a Based on information available from the RDP (release 11, update 4) and SILVA (release 123) databases. The numbers of sequences in each corresponding database are 3,333,501 (RDP, March 2016) and 1,756,783 (SILVA, March 2016). mism, mismatch(es). Reported are the numbers of sequences (hits) which theoretically hybridize with the probe, allowing for the given number of mismatches.

Additionally, to discriminate the symbiont observed in isolate US_Bl 11III1 from the already-described “Candidatus Fokinia solitaria,” the previously published probes Fokinia_198, Fokinia_434, and Fokinia_1250 (33) were tested *in silico* and in FISH experiments for their binding to the SSU rRNA of the second endosymbiont from isolate US_Bl 11III1.

### Phylogenetic analyses.

Phylogenetic analyses were performed with the ARB software package version 5.2 (38). The prokaryotic SSU rRNA gene sequences of the endosymbiont present in both isolates were aligned with 18 sequences of the RAM clade (six sequences of each family: Rickettsiaceae, Anaplasmataceae, and “Candidatus Midichloriaceae”), 68 sequences of “basal Rickettsiales” (20 sequences belonging to Holospora and related organisms, 15 sequences of Caedibacter-like organisms, 25 representative sequences of “Candidatus Paracaedibacteraceae,” and eight sequences associated with “Candidatus Hepatincola”). Seven representatives of other Alphaproteobacteria orders were chosen as the outgroup (data set 1). A second set of sequences (data set 2) was chosen to analyze the prokaryotic SSU rRNA gene sequence of the second endosymbiont of isolate US_Bl 11III1. The sequence was aligned with 23 other sequences of “Candidatus Midichloriaceae.” Nine sequences of Anaplasmataceae were chosen as an outgroup. For phylogenetic inference, alignment of both data sets was performed with the automatic aligner of the ARB software and further refined manually, to optimize stems and loops according to the predicted secondary structure of the prokaryotic SSU rRNA of E. coli. The alignments were trimmed at both ends to the shortest shared sequence, and gaps were treated as missing data. Additionally, for data set 1, hypervariable nucleotide positions (i.e., positions in which the most represented nucleotide was present in less than 20% of the organisms) were left out.

The optimal substitution model for each alignment was selected with jModelTest 2.1 (39), using the Akaike Information Criterion (AIC). Maximum likelihood (ML) trees were calculated with 1,000 bootstrap pseudoreplicates within the PhyML software, version 2.4.5, from the ARB package (40). Bayesian inference (BI) was performed with MrBayes 3.2 (41), using three runs each, with one cold and three heated Markov chain Monte Carlo runs, iterated for 1,500,000 generations. Sampling was performed every 500 generations with a burn-in of 25%. The recommendations of the MrBayes manual were followed to determine that convergence was reached.

### Transmission electron microscopy (TEM).

Paramecium cells of both isolates were fixed as described by Szokoli and colleagues (33) and embedded in epoxy embedding medium (Fluka Chemie AG, BioChemika, St. Gallen, Switzerland) according to the manufacturer's protocol. The blocks were sectioned with a Leica EM UC6 Ultracut microscope. Sections were stained with aqueous 1% uranyl acetate followed by 1% lead citrate. All samples were examined with a JEM-1400 electron microscope (JEOL, Ltd., Tokyo, Japan) at 90 kV. The images were obtained with a built-in digital camera.

### Accession number(s).

The joined sequences of the eukaryotic SSU, ITS, and partial LSU for each of the two isolates of P. biaurelia were submitted to NCBI GenBank and assigned accession numbers KU729876 and KU729877. Moreover, both COI gene sequences were submitted and assigned the accession numbers KX008305 and KX008306. Furthermore, nearly full-length prokaryotic SSU rRNA gene sequences of “Candidatus Bealeia paramacronuclearis” inhabiting the two isolates and of the second symbiont of isolate US_Bl 11II1, “Candidatus Fokinia cryptica,” were submitted to NCBI GenBank and assigned accession numbers KU736844, KU736845, and KU736846.

## RESULTS

### Characterization of the host.

General morphological features of the isolates, including body size and shape, number and shape of micronuclei, and location of the cytoproct, were typical for representatives of the Paramecium aurelia complex. Nearly full-length eukaryotic SSU rRNA gene sequences of both isolates were identical (both 1,711 bp), whereas ITS regions, including 5.8S and a part of the LSU rRNA gene, showed one nucleotide difference, located in the partial 28S rRNA gene sequence. The joined sequences of the eukaryotic SSU, ITS, and partial LSU for each of the two isolates were submitted to NCBI GenBank (2,800 bp). Eukaryotic SSU rRNA gene and ITS sequences supported both isolates belonging to the Paramecium aurelia group. Sequence identity with respect to other published ITS sequences of the P. aurelia complex ranged between 98 and 100% and dropped to 94 to 95% for Paramecium caudatum and Paramecium multimicronucleatum. Generally, COI gene sequences showed a higher resolution power than eukaryotic SSU rRNA gene and ITS regions. Thus, partial COI genes of isolates US_Bl 11III1 and US_Bl 15I1 were sequenced (760 bp). No differences between the two sequences were observed. Their sequence identities to Paramecium biaurelia sequences ranged from 99 to 100%, whereas identities with other members of the P. aurelia complex dropped to 80 to 85%. Therefore, based on COI gene sequences and in accordance with morphological features, we identified our isolates as P. biaurelia.

### Molecular characterization of “Candidatus Bealeia paramacronuclearis.”

**FIG 1:**
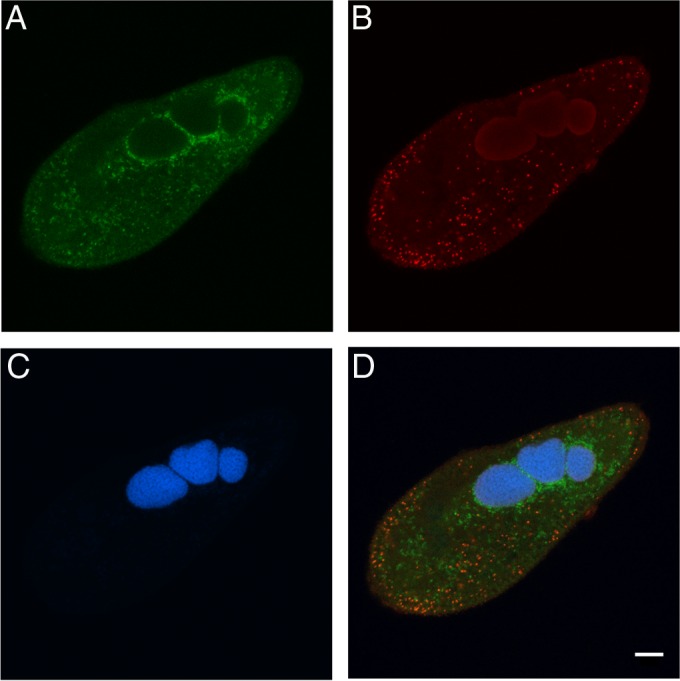
Species-specific *in situ* detection of “Candidatus Bealeia paramacronuclearis.” (A and B) Green fluorescent signal from probe Bealeia_1245 (A) and “Candidatus Fokinia cryptica” red fluorescent signal from probe FokCry_198 (B) in Paramecium biaurelia isolate US_Bl 11III1, both with 0% formamide. (C and D) 4′,6-Diaminidino-2-phenylindole (DAPI) signal (C), also illustrated in a merged image of the Bealeia_1245, FokCry_198, and DAPI signals (D). Relative positioning of both endosymbionts with respect to each other and within the host cell is shown. Scale bar, 10 μm.

The endosymbiont inhabiting isolates US_Bl 11III1 and US_Bl 15I1 represents a new bacterial species and genus and was designated “Candidatus Bealeia paramacronuclearis.” Bacterial SSU rRNA gene sequences of the endosymbionts from P. biaurelia US_Bl 11III1 and US_Bl 15I1 were identical through their entire length (1,445 bp). The species-specific probe Bealeia_1245 hybridized with its target organism in formamide from 0 to 25% (vol/vol) (Fig. 1), with the signal intensity being higher between 0 and 10% (vol/vol) formamide. In the *in silico* tests, probe specificity was higher for zero mismatches, but 179 nontarget sequences were recognized when one mismatch was allowed (Table 1). Comparing the obtained sequences with those available from NCBI GenBank, the highest sequence identity (97.1%) was observed with uncultured environmental bacteria retrieved from marine or extreme habitats (e.g., an acid mine drainage site) and from a wastewater treatment plant (accession numbers HQ420145, KJ782860, JN671986, and DQ988310). The described bacterial endosymbionts showing a higher sequence identity (range, 88 to 89%) were Caedibacter caryophilus (accession number NR_044847) from the macronucleus of Paramecium caudatum (42) and “Candidatus Nucleicultrix amoebiphila” (accession number KF697195) infecting the nucleus of an amoeba (43).

**FIG 2:**
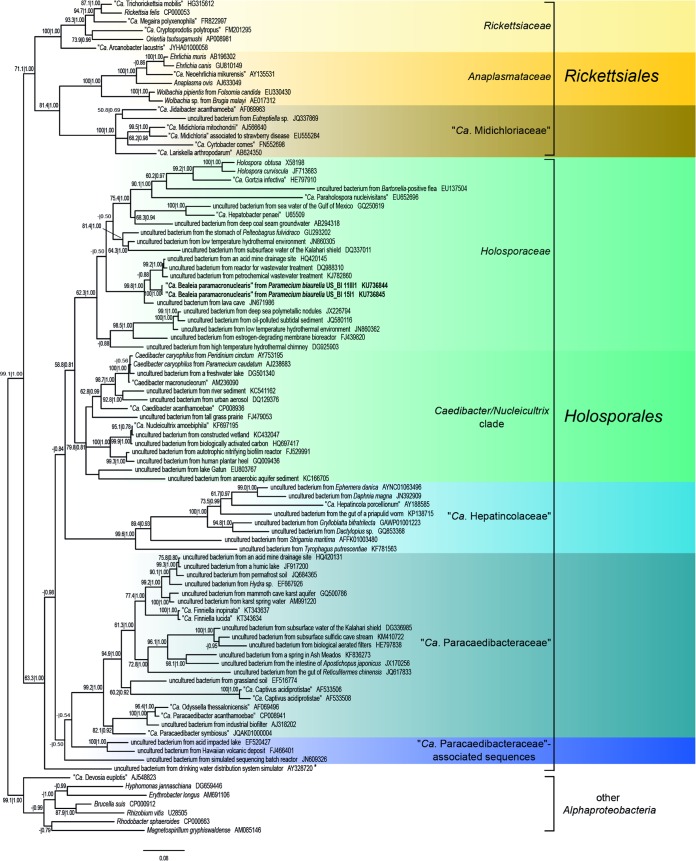
Bayesian inference phylogenetic tree of “Candidatus Bealeia paramacronuclearis,” based on prokaryotic SSU rRNA gene sequences, including 18 Rickettsiales, 70 Holosporales, and 7 members of other orders of Alphaproteobacteria as an outgroup. The GTR+I+G model was employed. A total of 1,251 nucleotide columns were used to calculate the tree. Numbers indicate maximum likelihood bootstrap values after 1,000 pseudoreplicates and Bayesian posterior probabilities after 1,500,000 iterations. Values below 50% and 0.5 are not shown. Scale bar, 8 nucleotide substitutions per 100 positions. *Ca*., Candidatus. *, a sequence which clusters within “Candidatus Paracaedibacteraceae,” including associated sequences in the ML tree (see Fig. 3).

**FIG 3:**
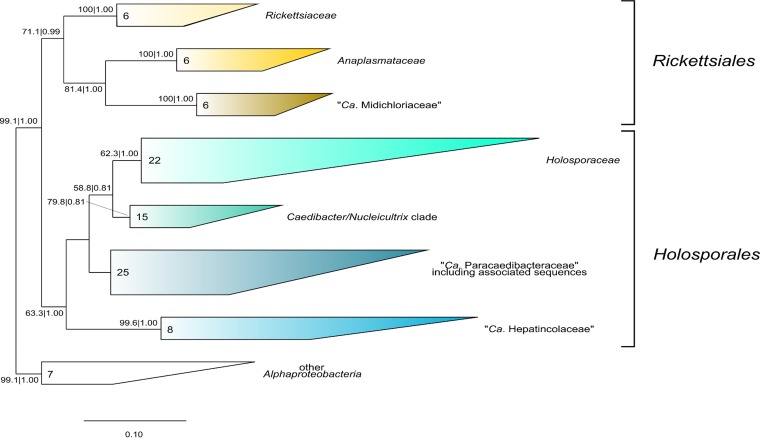
Phylogenetic tree topology of the maximum likelihood analysis employing the GTR+I+G model, showing a different relationship between the families of the order Holosporales with respect to the Bayesian inference tree (Fig. 2). Species selection and nucleotide columns used to calculate the tree are the same as those for the tree in Fig. 2. Numbers indicate maximum likelihood bootstrap values after 1,000 pseudoreplicates and Bayesian posterior probabilities after 1,500,000 iterations (values below 50% and 0.5 are not shown). Scale bar, 10 nucleotide substitutions per 100 positions. *Ca*., Candidatus.

In our phylogenetic analyses (Fig. 2 and 3), “Candidatus Bealeia paramacronuclearis” was always associated with the previously mentioned uncultured bacteria sequences, forming a genus-level “Candidatus Bealeia” clade (99.8% bootstrap support for ML and posterior probability of 1.00 for BI). All tree topologies obtained supported an association of “Candidatus Bealeia” with the genera Holospora, “Candidatus Gortzia,” “Candidatus Paraholospora,” and “Candidatus Hepatobacter” and with several sequences from uncultured organisms in a rather well-supported clade (62.3% bootstrap for ML and posterior probability of 1.00 for BI), for which we propose a family-level status and the name Holosporaceae. Another family-level clade (here called the Caedibacter-Nucleicultrix clade), containing endosymbiont species such as Caedibacter caryophilus and “Candidatus Nucleicultrix,” has sufficient support (79.8% for ML and 0.81 for BI) and branched as a sister group to Holosporaceae. The recently introduced family “Candidatus Paracaedibacteraceae” of Hess and colleagues (44) was confirmed by our analyses. A small group of sequences from uncultured organisms (EF520427, FJ466401, JN609326, and AY328720) was unstably associated with “Candidatus Paracaedibacteraceae” (Fig. 2 and 3), but their positioning could not be unambiguously resolved (i.e., association had low support and in BI analysis [Fig. 2]) (sequence AY328720 branches independently from “Candidatus Paracaedibacteraceae”). A well-supported clade (99.6 for ML and 1.00 for BI) of putative fast-evolving bacterial sequences associated with different metazoan organisms, including “Candidatus Hepatincola porcellionum,” could additionally be retrieved. Phylogenetic positioning of this clade was unstable, being sister to all other previously mentioned clades (Holosporaceae, Caedibacter-Nucleicultrix clade, “Candidatus Paracaedibacteraceae”) in ML (Fig. 3), or sister to only the Holosporaceae and Caedibacter-Nucleicultrix clades in BI trees (Fig. 2). We also propose a family-level status for this clade, with the name “Candidatus Hepatincolaceae,” referring to the first-described endosymbiont of this clade, “Candidatus Hepatincola porcellionum” (45). Our tree topologies show a separation of “traditional” Rickettsiales into two major groups: Rickettsiaceae, Anaplasmataceae, and “Candidatus Midichloriaceae” (RAM clade) (reported in Fig. 2 and 3 as Rickettsiales) and Holosporaceae, Caedibacter-Nucleicultrix clade, “Candidatus Paracaedibacteraceae,” and “Candidatus Hepaticolaceae” (reported in Fig. 2 and 3 as Holosporales).

The prokaryotic SSU rRNA gene sequence identities within the RAM clade on one side, and within the clade including Holosporaceae, the Caedibacter-Nucleicultrix clade, “Candidatus Paracaedibacteraceae,” and “Candidatus Hepaticolaceae” on the other side, were compared with the order values retrieved by Yarza and coauthors (46). The identities were on average higher than the threshold (86.3% and 85.7%, respectively), although in both groups some values, always associated with fast-evolving organisms, were found below the threshold. Conversely, the average identity between the groups was comparatively lower (82.8%) (see Table S1 in the supplemental material).

### Molecular characterization of “Candidatus Fokinia cryptica.”

The second bacterial SSU rRNA gene sequence, derived only from isolate US_Bl 11III1 (1,454 bp), was found to be closely related to “Candidatus Fokinia solitaria” (33), as it shared 97.7% sequence identity. Thus, we propose the name “Candidatus Fokinia cryptica,” due to the difficulties we had in molecularly characterizing this endosymbiont. Both SSU rRNA genes share short insertions at positions 76, 94, 200, and 216 (according to E. coli SSU rRNA gene reference numbering) whose sequences slightly differ.

Given the “Candidatus Fokinia cryptica” sequence, the two previously published probes Fokinia_434 and Fokinia_1250 (33) turned out to be specific for the “Candidatus Fokinia” genus. In FISH experiments, using one of these probes in combination with EUB338, a positive and overlapping signal was obtained for a subset of the endosymbiotic bacteria in P. biaurelia isolate US_Bl 11III1, whereas others (endosymbionts and food bacteria inside food vacuoles) were targeted only by EUB338. Similar experiments, in which genus-specific “Candidatus Fokinia” probes were used in combination with the Bealeia_1245 probe, showed that the “Candidatus Fokinia” and “Candidatus Bealeia” probes targeted different symbionts within the same host cell without any overlap (Fig. 1). The previously published probe Fokinia_198 turned out to be specific for the previously published “Candidatus Fokinia solitaria” species and has been consequently renamed FokSol_198; this expectation was confirmed by negative FISH results with probe FokSol_198 against isolate US_Bl 11III1 bearing “Candidatus Fokinia cryptica” (data not shown). The species-specific probe FokCry_198, targeting the same region, was designed for “Candidatus Fokinia cryptica” and was validated to have a high specificity (Table 1). Its optimal performance is at 0 to 30% (vol/vol) formamide, but it gives a signal with up to 50% formamide. It does not hybridize to “Candidatus Fokinia solitaria” at any stringency (data not shown).

**FIG 4:**
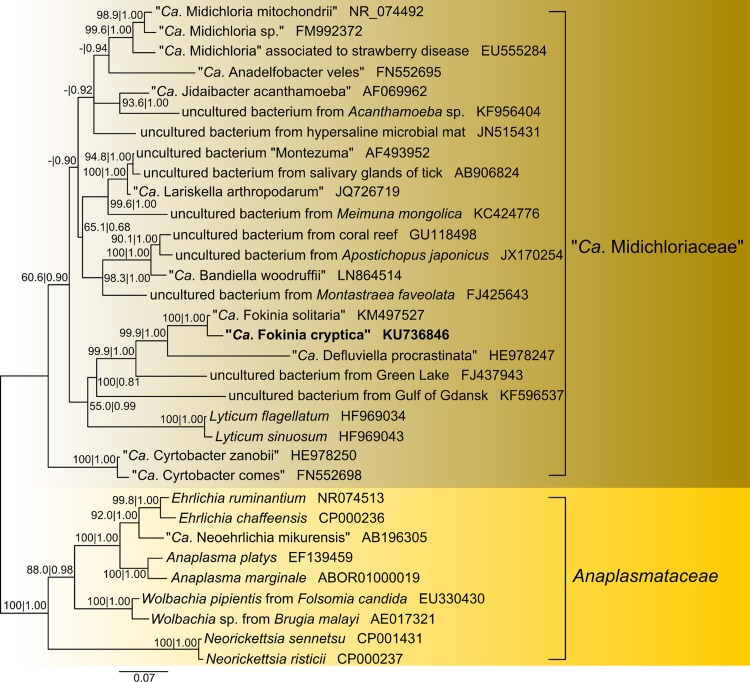
Bayesian inference phylogenetic tree (GTR+I+G model) of “Candidatus Fokinia cryptica,” based on prokaryotic SSU rRNA gene sequences, including 23 other “Candidatus Midichloriaceae” and 9 Anaplasmataceae as an outgroup. Nucleotide columns used to calculate the tree: 1,311. Maximum likelihood bootstrap values after 1,000 pseudoreplicates and Bayesian posterior probabilities after 1,500,000 iterations are shown down to 50% and 0.5. Scale bar: 7 nucleotide substitutions per 100 positions. *Ca*., Candidatus.

The phylogenetic position of “Candidatus Fokinia cryptica” is clearly inside the family “Candidatus Midichloriaceae” (Rickettsiales, Alphaproteobacteria) and associated with high support values by both inference methods (100% for ML and 1.00 for BI) to “Candidatus Fokinia solitaria” (Fig. 4). Both sequences clustered with “Candidatus Defluviella procrastinata,” colonizing Paramecium nephridiatum (accession number HE978247), and an environmental sequence isolated from Green Lake, NY (accession number FJ437943).

### Ultrastructural observations.

**FIG 5:**
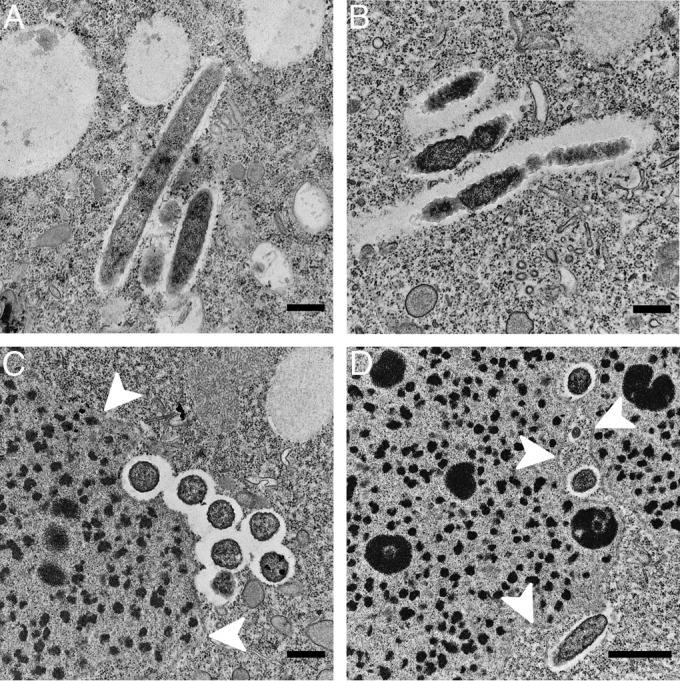
Transmission electron microscopic images of “Candidatus Bealeia paramacronuclearis,” as observed for Paramecium biaurelia isolates US_Bl 11III1 and US_Bl 15I1. The symbiotic bacteria are oriented in parallel, in groups (A, B, and C), and were often found associated with the macronucleus (C and D), sometimes even lying in its folds (D). White arrowheads indicate the nuclear envelope. Scale bars, 0.5 μm (A, B, and C) or 1.0 μm (D).

In electron micrographs of P. biaurelia isolate US_Bl 15I1, only one kind of endosymbiont was observed in the cytoplasm. Symbionts had the two typical membranes of Gram-negative bacteria, reached 1.8 to 2.4 by 0.4 to 0.5 μm in size, and were devoid of flagella. The endosymbionts had an electron-dense cytoplasm (Fig. 5A) with conspicuous ribosomes and nucleoid. No host membrane enclosing these bacteria was observed, but they were always surrounded by an electron-lucid halo lacking host ribosomes. These endosymbionts often formed clusters of up to 7 or 8 cells, with parallel orientation (Fig. 5A and B). The bacteria tended to locate close to the Paramecium macronucleus and could sometimes be found in deep folds of the nuclear envelope, which nevertheless remained intact (Fig. 1 and 5C and D), hence the proposed species name, “Candidatus Bealeia paramacronuclearis.”

**FIG 6:**
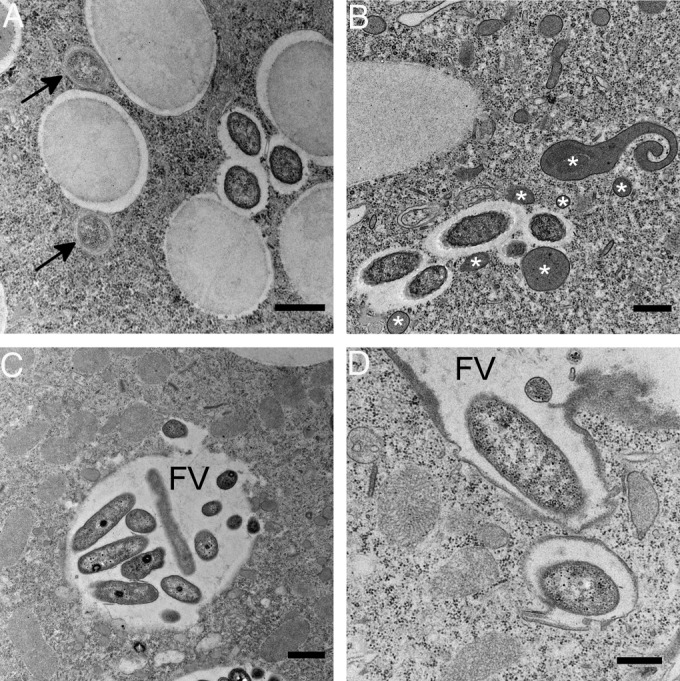
Transmission electron microscopic images of Paramecium biaurelia isolate US_Bl 11III1. (A) For this isolate, the secondary symbiont “Candidatus Fokinia cryptica,” indicated by black arrows, was observed in cooccurrence with “Candidatus Bealeia paramacronuclearis.” (B) “Candidatus Bealeia paramacronuclearis” was frequently surrounded by host lysosomes (white asterisks). (C and D) Parts of the host's digestive vacuole (food vacuole [FV]) containing bacteria morphologically similar to “Candidatus Bealeia paramacronuclearis” were seen to bud off, producing vesicles enclosing them. Scale bars, 0.5 μm (A, B, and D) or 1.0 μm (C).

In electron micrographs of isolate US_Bl 11III1, some bacteria showed an identical morphology and cytoplasmic distribution and were attributed to the species “Candidatus Bealeia paramacronuclearis.” In this host, a second type of bacterium, with an electron-lucid cytoplasm, was evident. These cells (size, 1.1 by 0.35 to 0.40 μm) were never found in clusters, were enclosed in a symbiontophorous vacuole (Fig. 6A), and resembled “Candidatus Fokinia solitaria” (33). They tended to locate in the trichocyst layer (Fig. 6A), which was also seen via FISH observations, but could occasionally be observed in other regions of the cytoplasm.

Host lysosomes were often seen near “Candidatus Bealeia paramacronuclearis,” located in the endoplasm (Fig. 6B). In the isolate US_Bl 11III1, digestive vacuoles looked highly unusual, forming evaginations containing bacteria, supposedly “Candidatus Bealeia paramacronuclearis.” These evaginations were seen to bud off, producing vesicles enclosing the bacteria. Nothing of the kind is normally observed in TEM sections of endosymbiont-free paramecia fed Raoultella, or in the other isolate. Interestingly, “Candidatus Bealeia paramacronuclearis” encircled by a host membrane could be found only in the vicinity of food vacuoles, suggesting that the host membrane was later broken (Fig. 6C and D).

## DISCUSSION

### Disentangling the inner phylogenetic relationships of Rickettsiales.

The phylogenetic relationships inside the order Rickettsiales are at present much debated (10–12, 44, 47, 48). The families Rickettsiaceae, Anaplasmataceae, and “Candidatus Midichloriaceae” (RAM clade) represent highly supported monophyletic clades (12–19). The fourth family, Holosporaceae (49), formerly joined all remaining species of Rickettsiales, such as Holospora (50), Caedibacter caryophilus (42), “Candidatus Paracaedibacter symbiosus” (51), “Candidatus Odyssella thessalonicensis” (52), “Candidatus Captivus acidiprotistae” (53), “Candidatus Hepatincola porcellionum” (45), “Candidatus Paraholospora nucleivisitans” (54), “Candidatus Gortzia infectiva” (47), and “Candidatus Hepatobacter penaei” (55) and sequences of uncultured bacteria closely related to these species. Recent analyses mainly based on alternative genetic markers (10–12, 48) provided a different and more complex perspective in which Holosporaceae is not a sister group of other Rickettsiales.

Boscaro and colleagues (47) suggested that the true family Holosporaceae (sensu stricto) consisted solely of Holospora spp. and Holospora-like bacteria (HLB), such as “Candidatus Gortzia infectiva,” while other species should be placed inside Rickettsiales as incertae sedis. With the ongoing accumulation of data, we believe that this Holosporaceae family definition is too strict. Indeed, it would exclude all phylogenetically related species lacking the typical Holospora life cycle. Although subfamily rank is seldom used nowadays in bacterial taxonomy, we suggest establishment of the subfamily Holosporoidea, to include Holospora spp. and Holospora-like bacteria with the typical features mentioned above; Holosporoidea *sensu* Szokoli (this study) would correspond to Holosporaceae *sensu* Boscaro and colleagues (47). Consequently, applying a phylogenetic approach, we propose to include in the family Holosporaceae the following: “Candidatus Paraholospora nucleivisitans,” “Candidatus Hepatobacter penaei,” “Candidatus Bealeia paramacronuclearis,” and related sequences of uncultured organisms.

A sister clade to Holosporaceae, comprising Caedibacter caryophilus, “Candidatus Nucleicultrix,” and further environmental sequences, emerged in our analyses. This clade has been already retrieved in previous studies (12, 44), and its formal description, which is rather complex due to taxonomic issues, is ongoing but not yet published (M. Schrallhammer et al., unpublished data).

The family “Candidatus Paracaedibacteraceae” has recently been proposed, joining endosymbiotic bacteria such as “Candidatus Paracaedibacter symbiosus,” “Candidatus Odyssella thessalonicensis,” “Candidatus Captivus acidiprotistae,” the novel genus “Candidatus Finniella,” and other environmental sequences (44). Based on our analyses, we confirmed the family level of the clade, whose borders in the future could possibly be broadened with respect to the available species composition.

Depending on the phylogenetic method employed, the well-supported clade “Candidatus Hepatincolaceae,” represented by bacteria associated with Ecdysozoa hosts, falls at different positions in our analyses: either as sister to the Holosporaceae and Caedibacter-Nucleicultrix clade (Fig. 2; based on BI) or as sister to the Holosporaceae-Caedibacter-Nucleicultrix-Paracedibacteraceae clade (Fig. 3; based on ML). Their rRNA gene sequences seem to be fast evolving, and thus a precise placement within the groups will require further analyses.

Our phylogenies show a separation of the traditional Rickettsiales into two major groups, which would deserve the order level: one containing Rickettsiaceae, Anaplasmataceae, and “Candidatus Midichloriaceae” (RAM clade), and one consisting of “basal Rickettsiales.” Among the sequences included in our analysis, several display high evolutionary rates, especially those derived from environmental origin. They were included in order to get a comprehensive and representative selection of “basal Rickettsiales” and its subclades. As a probable and unfortunate consequence, the presence of such fast-evolving sequences can also produce instabilities in phylogenetic reconstructions and is likely causing relatively lower support values of some branches in the ML tree (Fig. 2) compared to other previously published studies (11, 17, 44, 47, 48).

The members of each major group display a wide range of identity values in their prokaryotic SSU rRNA genes (see Table S1 in the supplemental material). Within each group, the average identities are above the threshold retrieved by Yarza and colleagues (46), limiting orders, although some values were found below this threshold. These unusual values contribute to the high variability present within each group and can be attributed as well to the numerous SSU rRNA sequences with high evolutionary rates.

Taking into account what we have reported here, we propose to establish the order Holosporales ord. nov. (according to Ferla et al. [11]), including the four family-level clades Holosporaceae, the Caedibacter-Nucleicultrix clade, “Candidatus Paracaedibacteraceae” including associated sequences, and “Candidatus Hepatincolaceae.” As previously anticipated, further analyses are needed to clarify both the internal relationships within the order Holosporales (in particular for what concerns “Candidatus Hepatincolaceae”) and the relationship of Holosporales with respect to the Rickettsiales sensu stricto herein defined and to all other Alphaproteobacteria in general. Nevertheless, we emphasize that to our best knowledge the vast majority of published analyses (all except the study by Hess and colleagues [44]) agree among themselves and with us on the delineation of Holosporales as a monophyletic clade, therefore offering adequate support for its elevation to the order rank. Additionally, although the available data on Holosporales (other than 16S rRNA gene sequences) are overall much scarcer than those for Rickettsiales sensu stricto, some quite well-defined differences are already evident. From an ecological point of view, the host range of described organisms appears to include, with a much higher prevalence, aquatic organisms (55) and in particular protists (e.g., 12, 43, 44, 49, 52, 53). Also, at a genomic level, the nine genome sequences currently available for Holosporales via NCBI are overall significantly larger (1.1 to 2.85 Mb) than typical Rickettsiales sensu stricto sequences (mostly in the range 1 to 1.5 Mb) (9). Rickettsiales sensu scricto have been studied quite extensively for the mechanisms of interaction with the host, such as secretion systems and effectors (56–59). On the contrary, even in model organisms such as Holospora and Caedibacter (60, 61), Holosporales are still poorly investigated in this respect. Hopefully, future studies providing additional data will offer new insights on the features of Holosporales and the differences with Rickettsiales sensu stricto.

### Life in a triangular relationship.

Only a few reports of multiple infections of ciliates are available in the literature. Spirostomum minus (Heterotrichea) was found with bacteria colonizing both the cytoplasm and the macronucleus (20, 22). Euplotes spp. (Spirotrichea) infected by Polynucleobacter necessarius and other bacteria have been reported as well (16, 24, 62); the Euplotes aediculatus isolate In from New Delhi (India) bore three different cytoplasmic endosymbionts (18, 29, 63) beyond P. necessarius (64). In Paramecium, multiple infections often occur in combination with infectious Holospora species inhabiting the macro- or micronucleus of P. caudatum, or even in combination with other cytoplasmic bacteria. Some authors have suggested that Holospora can act as a vector for other bacteria, including food bacteria, facilitating their entrance into the host (20, 65). On the contrary, the presence of symbiotic chlorellae in P. bursaria is generally considered protective against infections by other cytoplasmic endosymbionts; nevertheless, several reports are available of bacterial endosymbionts coexisting with those symbiotic algae in the same P. bursaria host, such as “Holospora acuminata” (reviewed in reference 21), Pseudocaedibacter chlorellopellens (7, 60), and “Candidatus Sonnebornia yantaiensis” (66).

It has been suggested that if endosymbionts colonize different compartments, they probably depend on different metabolites and, therefore, do not compete directly with each other (21). The interactions among the host and its symbionts perhaps become more complicated if the same cell compartment is colonized. Another S. minus isolate bore five different symbiotic bacteria, of which three were found in the cytoplasm (20, 22). The symbionts were lost during the maintenance of the isolate under laboratory conditions, indicating the instability of these symbiotic associations. Multiple infections are probably more frequent than we know, but due to competition and other factors (e.g., stress reaction and/or host defense mechanisms), only a few have been observed and even fewer are stable under laboratory conditions over time. In the cases of stable infections, the different endosymbionts may be in a balanced competition with each other, resulting in their persistent cooccurrence. A different explanation was proposed by Görtz (21), who suggested that the bacteria could have favorable effects on each other. It is not clear whether “Candidatus Bealeia paramacronuclearis” and “Candidatus Fokinia cryptica” interact in a beneficial way with each other or with their host, or if they live in balanced competition. However, they appear to be adapted to their three-partner symbiotic system and do not necessarily compete for resources. This could be a reasonable explanation for the long-term stability of their double infection. Future analyses, possibly performed on (meta-)genomic sequences, could help in clarifying the role of each symbiont and the interactions among them and with the host.

“Candidatus Bealeia paramacronuclearis” was often found in association with the host macronucleus. It is intriguing to observe that most bacteria described within Holosporaceae (*sensu* in this report) show nuclear forms or at least nucleus-associated forms. Referring to the close association of “Candidatus Bealeia paramacronuclearis” with the host's macronucleus, we propose the name “Candidatus Bealeia paramacronuclearis,” in accordance with the guidelines of the International Committee on Systematic Bacteriology (67). Furthermore, we honor Geoffrey Herbert Beale (11 June 1913 to 16 October 2009), a prominent British geneticist who did seminal work on Paramecium and its symbionts.

Electron micrographs of “Candidatus Bealeia paramacronuclearis” suggest the possibility that the bacterium is able to escape food vacuoles by deforming the membrane and disassociating a portion from the main food vacuole (Fig. 6C and D). Later, the bacterium seems to escape from the host vacuole, since we never find it enclosed by a membrane when in the cytoplasm. Unfortunately, no morphologically distinguishable infectious forms were observed, and preliminary infection experiments using cell lysates, similar to those used for Holospora (modified from the methods described by Magalon et al. [68]) have so far failed (data not shown). Despite the absence of experimental evidence, the occurrence of four sequences closely related to “Candidatus Bealeia paramacronuclearis” in different types of habitats (marine, wastewater, and extreme habitats) suggests that this bacterium might be horizontally transferred, as for Holospora and “Candidatus Gortzia infectiva” (47, 61).

“Candidatus Fokinia cryptica” showed several similarities to the formerly described species “Candidatus Fokinia solitaria” (33), not only on a molecular level but also morphologically: both species appeared as very small, electron-lucid bacteria in electron micrographs. However, whereas “Candidatus Fokinia solitaria” was restricted to a narrow layer at the host cortex, probably avoiding host defense mechanisms (33), “Candidatus Fokinia cryptica” was not observed strictly in the cortex but occasionally also in the host endoplasm. Moreover, in contrast to “Candidatus Fokinia solitaria,” “Candidatus Fokinia cryptica” showed a host-derived symbiontophorous vacuole, which is probably protective against xenophagy, thus allowing the broader distribution in the host endoplasm. Interestingly, the fine structure of “Candidatus Fokinia cryptica” and the presence of the symbiontophorous vacuole resembled the descriptions of Gamma particles, later denominated Pseudocaedibacter minutus from Paramecium octaurelia, first described by Preer and coauthors (69). However, in the latter case, the membrane surrounding the endosymbiont was densely covered with ribosomes and the host demonstrated a strong killer effect. Any reference cell lines bearing Pseudocaedibacter minutus are no longer available from culture collections, and so this issue will remain open.

### Conclusions.

Observations of double or multiple infections of ciliates by endosymbionts are relatively rare. Only a few reports are available (16, 20–24). In P. biaurelia isolate US_Bl 11III1, we have found a stable double infection of novel endosymbiotic bacteria. From our observations, both “Candidatus Bealeia paramacronuclearis” and “Candidatus Fokinia cryptica” occur together in the host's cytoplasm—now for 4 years under laboratory conditions—and hence live in stable association within the same host compartment. Thus, the bacteria may be different in their metabolic requirements, and their cooccurrence must not harm Paramecium to the extent that extinction of either the host or one of its symbionts occurs.

Our phylogenetic results support the appropriateness of separating all “basal Rickettsiales” from the order Rickettsiales sensu stricto to form the new order Holosporales ord. nov. (according to Ferla et al. [11]). Consequently, the order Rickettsiales now consists solely of the RAM clade families Rickettsiaceae, Anaplasmataceae, and “Candidatus Midichloriaceae.” Furthermore, we suggest broadening the borders of the family Holosporaceae, as defined by Boscaro and coworkers (47), by including the genera “Candidatus Paraholospora,” “Candidatus Hepatobacter,” and “Candidatus Bealeia,” as well as all associated environmental sequences. Moreover, we identified a new family-level clade that includes the “Candidatus Hepatincola porcellionum” sequence, which we have named “Candidatus Hepatincolaceae.” Members of this clade show elevated evolutionary rates in their rRNA gene sequences, resulting in an unstable positioning within Holosporales. Genome analyses of our novel endosymbiont species and other members of the orders Rickettsiales and Holosporales will be necessary to unambiguously resolve the phylogenetic position of these two orders and to reveal the secrets of multiple endosymbiont-host interactions.

### Emended description of “Candidatus Fokinia” Szokoli et al. (2016).

“Candidatus Fokinia” (Fo.ki'ni.a so.li. ta'ri.a; N.L. fem. n. Fokinia, in honor of Sergei I. Fokin). The description of “Candidatus Fokinia” (33) is emended as follows: FISH experiments confirmed the genus specificity of the oligonucleotide probes Fokinia_434 and Fokinia_1250 (33), with both known species of the genus, “Candidatus Fokinia solitaria” (type species) and “Candidatus Fokinia cryptica.”

### Description of “Candidatus Fokinia cryptica” sp. nov.

“Candidatus Fokinia cryptica” (cryp'ti.ca. N.L. fem. n. kryptikós hidden). Short rod-like bacterium (1.1 by 0.35 to 0.40 μm in size). Cytoplasmic endosymbiont of the ciliate Paramecium biaurelia (Oligohymenophorea, Ciliophora), residing in symbiontophorous vacuoles. Basis of assignment: SSU rRNA gene sequence (accession number KU736846) and a positive match with the specific FISH oligonucleotide probe FokCry_198 (5′-CTCGCAGTAACATTGCTGC-3′). Belongs to “Candidatus Midichloriaceae” family, order Rickettsiales, class Alphaproteobacteria. Identified in Paramecium biaurelia isolate US_Bl 11III1 from a small pond in the Miller Showers Park in Bloomington, IN. Uncultured thus far.

### Description of “Candidatus Bealeia” gen. nov.

“Candidatus Bealeia” (Bea'lei.a. N.L. fem. n. Bealeia named in honor of Geoffrey Herbert Beale (11 June 1913 to 16 October 2009).

The new genus encompasses endosymbiotic bacteria associated with the ciliate Paramecium biaurelia. Belongs to the family Holosporaceae within the order Holosporales, class Alphaproteobacteria. The type species of the genus is “Candidatus Bealeia paramacronuclearis.”

### Description of “Candidatus Bealeia paramacronuclearis” sp. nov.

“Candidatus Bealeia paramacronuclearis” (pa.ra.ma.cro.nu.cle.a'ris. Gr. prep. *para*, beside; L. masc. n. macronucleus vegetative nucleus of ciliates; N.L. fem. n. paramacronuclearis occurring beside the macronucleus).

Cytoplasmic endosymbiont of the ciliate Paramecium biaurelia (Oligohymenophorea, Ciliophora). Rod-like bacterium (1.8 to 2.4 μm by 0.4 to 0.5 μm in size) with an electron-dense cytoplasm and conspicuous ribosomes and nucleoid. Devoid of flagella. Often forms clusters of up to 7 or 8 cells, generally in close proximity to the host macronucleus. Basis of assignment: SSU rRNA gene sequence (accession number: KU736844) and positive match with the species-specific FISH oligonucleotide probe Bealeia_1245 (5′-CCTATTGCTTCCTTTTGTCAC-3′). Identified in Paramecium biaurelia isolate US_Bl 11III1 from a small pond in the Miller Showers Park in Bloomington, IN, and isolate US_Bl 15I1 from Yellowwood Lake (John Floyd Hollow, IN). Type strain is US_Bl 11III1. Thus far, uncultured.

### Emended description of the family Holosporaceae Görtz and Schmidt (2005).

Holosporaceae (Ho.lo.spo.ra'ce.ae. N.L. fem. n. Holospora type genus of the family; suff. -*aceae* ending to denote a family; N.L. fem. pl. n. Holosporaceae the family of genus Holospora).

According to phylogenetic analysis, the description of the family Holosporaceae is emended. Basis of assignment to this family is a stable clustering in phylogenetic trees with the type genus Holospora and not with the Caedibacter-Nucleicultrix clade; showing a prokaryotic SSU rRNA identity with Holospora higher than 81%. All described members of this family are obligate intracellular bacteria. Several possess two morphologically distinct forms and/or life cycles. The family currently comprises the genera Holospora, “Candidatus Gortzia,” “Candidatus Paraholospora,” “Candidatus Hepatobacter,” “Candidatus Bealeia,” and several sequences from uncultured organisms. Type genus is Holospora (49).

### Description of “Candidatus Hepatincolaceae” fam. nov.

“Candidatus Hepatincolaceae” (He.pat.in.co.la'ce.ae, N.L. fem. n. “*Ca*. Hepatincola” type genus of the family; suff. -*aceae* ending to denote a family; N.L. fem. pl. n. “Candidatus Hepatincolaceae” the family of genus “Candidatus Hepatincola”).

The family “Candidatus Hepatincolaceae” is defined based on phylogenetic analysis of SSU rRNA gene sequences of the type genus and uncultured representatives from various environments; most of the sequences are apparently associated with Ecdysozoa. The family currently contains one genus, “Candidatus Hepatincola” (45).

### Description of Holosporales ord. nov.

Holosporales (Ho.lo.spo.ra'les. N.L. fem. n. Holospora type genus of the order; suff. -*ales* ending to denote order; N.L. fem. pl. n. Holosporales the order of genus Holospora).

Defined by phylogenetic analyses based on SSU rRNA gene sequences. The order contains three families (Holosporaceae, “Candidatus Paracaedibacteraceae,” and “Candidatus Hepatincolaceae”), and the Caedibacter-Nucleicultrix clade, including Caedibacter caryophilus, Caedibacter varicaedens, “Caedibacter macronucleorum” (all endosymbionts of Paramecium species), “Candidatus Nucleicultrix amoebiphila” (endosymbiont of Hartmannella sp.), and several uncultured sequences. The order Holosporales is a member of the class Alphaproteobacteria. The type genus is Holospora.

## Supplementary Material

Supplemental material
