# Supplementary material for: Disentangling the Taxonomy of Rickettsiales and Description of Two Novel Symbionts (“Candidatus Bealeia paramacronuclearis” and “Candidatus Fokinia cryptica”) Sharing the Cytoplasm of the Ciliate Protist Paramecium biaurelia
Source: Appl Environ Microbiol. 2016 Nov 21;82(24):7236–47. doi: 10.1128/AEM.02284-16 (PMC5118934; doi:10.1128/AEM.02284-16)
Supplement: Supplemental material [file supp_82_24_7236__index.html]

Supplemental material 

# Disentangling the Taxonomy of Rickettsiales and Description of Two Novel Symbionts (“Candidatus Bealeia paramacronuclearis” and “Candidatus Fokinia cryptica”) Sharing the Cytoplasm of the Ciliate Protist Paramecium biaurelia

## Supplemental material

**Files in this Data Supplement:**

- Supplemental file 1 -

  Complete legend for Table S1.

  PDF, 175K
- Supplemental file 2 -

  Identity matrix of the sequences employed in the phylogeny shown in Fig. 3 (Table S1).

  XLS, 77K
