# Supplementary material for: Disentangling the Taxonomy of Rickettsiales and Description of Two Novel Symbionts (“Candidatus Bealeia paramacronuclearis” and “Candidatus Fokinia cryptica”) Sharing the Cytoplasm of the Ciliate Protist Paramecium biaurelia
Source: Appl Environ Microbiol. 2016 Nov 21;82(24):7236–47. doi: 10.1128/AEM.02284-16 (PMC5118934; doi:10.1128/AEM.02284-16)
Supplement: Supplemental material [file AEM.02284-16_zam999117574s1.pdf]

Supplementary table 1 Identity matrix of the sequences employed in the phylogeny shown in Figure 3. The two sequences of "*Candidatus* Bealeia paramacronuclearis" characterized in this study are reported in bold. The organism names and identities within *Rickettsiales sensu stricto* and *Holosporales* are reported in red and blue, respectively. The identities between *Rickettsiales sensu stricto* and *Holosporales* are reported in green. *Ca*. is an abbreviation for *Candidatus*. The self-identity of each sequence is reported in bold.
